# Personnel Training Course for Businesses Regarding the Response to Stranded Persons Focusing on Vulnerable People from the Perspective of Business Continuity

**DOI:** 10.3390/ijerph17124263

**Published:** 2020-06-15

**Authors:** Yuki Shibamura, Noriko Sudo, Gengaku Mashiro, Shigeru Beppu, Risa Hakamata, Kanata Saito

**Affiliations:** 1 Nutrition and Food Science Course, Faculty of Human Life and Environmental Sciences, Ochanomizu University, Tokyo 112-8610, Japan; g2040529@edu.cc.ocha.ac.jp (Y.S.); tan.kya7@gmail.com (K.S.); 2 Natural Science Division, Faculty of Core Research, Ochanomizu University, Tokyo 112-8610, Japan; 3 BC Research Institute, Tokyu Facility Service Co. Ltd., Tokyo 158-8539, Japan; gengaku.mashiro@tokyu-facility-service.co.jp (G.M.); risa.hakamata@tokyu-facility-service.co.jp (R.H.); 4 Food Processing Technology, Graduate School of Niigata University, Niigata City 950-2181, Japan; beep@bl.mmtr.or.jp

**Keywords:** stranded persons, vulnerable people, disaster training, business continuity

## Abstract

Businesses in urban areas have been required to accommodate stranded persons as temporary evacuation facilities during disasters. Regarding measures aimed at aiding stranded persons, aspects such as trust and the image of the business need to be considered. Therefore, in this study, a personnel training course was developed to smoothly take in stranded persons, and the outcomes of this training were evaluated by quizzes, entry sheets, and a questionnaire. This was a two-day and one-night course characterized by the use of role-play in which 20 participants experienced the series of processes that unfold during disasters, playing either the role of a stranded person or a facility member. This training included emergency food provision using real stockpiled food and accommodation training using actual bedding stored in a model facility. After the review, when the participants were taught the correct response for vulnerable people, their scores in the test regarding the points of caution in vulnerable people were significantly higher than those prior to the course, confirming that participants had acquired knowledge as a result. Furthermore, through training using real food and accommodation, the participants were able to understand victims’ requirements by experiencing the need for satisfactory emergency rations and comfortable bedding.

## 1. Introduction

The Great East Japan Earthquake that occurred on March 11, 2011 interrupted public transportation in the central Tokyo area, causing many stranded persons who were unable to get home from school, work, etc. [1]. Since the Great East Japan Earthquake, it has become mandatory for a business enterprise in Japan to function as a temporary shelter for those who are stranded in the event of a disaster [2]. The protection of stranded persons is important not only from a humanitarian aspect but also for the business after the disaster. Some enterprises improved their corporate image by providing their stockpiles or merchandise to support such people during the Great East Japan Earthquake and gained stock price increases afterward [3]. On the other hand, others faced severe criticism because closing their facilities for security reasons caused a massive increase in the number of people who were stranded [4,5]. Formulating a proper support plan for stranded people is a challenging task for many business enterprises, but it is an essential issue that directly affects their corporate image and trust and positively affects their post-disaster business performance.

Hence, business enterprises must suitably prepare and manage their stockpiles for the stranded and know how to provide support to vulnerable people, including the elderly, disabled, infants, pregnant women, or foreigners [6]. Previous incidents during similar events have shown the inadequacy of the services offered by the shelter management. For example, foreigners who were unable to communicate in Japanese did not receive sufficient assistance and refugees were not given proper meals because the management lacked knowledge in food allergies [7,8]. Advanced knowledge about the specific requirements will result in smooth customer service for those stranded during post-disaster disorder.

As stated above, a business enterprise needs to train and recruit personnel who understand the issues in receiving people who are stranded, including vulnerable people, and implement necessary managerial measures to promote its support plan. Several private and public organizations implement training programs for disaster events, for example, business corporations hold emergency drills throughout the year [9], municipal governments or schools implement shelter management exercises [10,11], and universities provide shelter simulation practice with lodging [12,13]. However, we need to establish a new foster program for business enterprises focused on the promotion of support measures for the stranded, including vulnerable people, during disaster events, and evaluate its educational effects.

In response to this, we have developed a foster program called “Simulation course for supporting disaster victims” (hereinafter referred to as the “course”) for business enterprises to implement their strategies per requirements. The course aims at cultivating a better understanding of supporting stranded people by learning through a series of role-play scenarios wherein the participants are divided into two groups, management staff and stranded people.

Disaster drills that simulate serious incidents and train staff how to respond to them are widely used [14]. Yoshida et al. developed a simulation system that provides a virtual experience of disaster and shares the recollections of stranded commuters [15]. Our course is not computer-based and includes emergency food provision using real stockpiled food and accommodation training using actual bedding stored in a model facility. It allows a participant to interact with others, causing a conflict of interests and stress that many refugees experience in mass care. Scenario methods are useful in developing skills such as decision-making under stress [16].

To evaluate the course’s effectiveness as a foster program for business enterprises, we aimed at (1) improving participants’ knowledge regarding vulnerable people and (2) organizing a list of commodities necessary for improving stockpiles in shelters. We held quizzes (see Appendix A) before and after the course, asked participants to fill in entry sheets regarding what they learned during the role-play, conducted a questionnaire survey after the course, and organized the results for evaluation.

## 2. Materials and Methods

### 2.1. The Course Outline

#### 2.1.1. The Course Contents

The course was held at the training center of Tokyu Facility Service Co., Ltd. for two days and one night from 1–2 August 2019. Table 1 shows the course items and their objectives.

#### 2.1.2. Position of the Course

The course was conducted as an optional program in the BC (business continuity; the capability of the organization to continue delivery of products or services at acceptable predefined levels following a disruptive incident) training program held by Tokyu Facility Service Co., Ltd. The BC training program aims at cultivating human resources for BC promotion in the organization. It consists of the core program called “Basic Course” wherein participants visit disaster sites for two days and one night and reflect on their BC practice for possible improvements through a series of interview sessions with managers of the firms affected by the disaster, along with three optional programs, including the course we are discussing in this study. If our course and the BC program are received together, it will enhance the training’s effect for the personnel who engage in the BC practice and promote supportive measures during disaster events.

### 2.2. Participants and Setting

#### 2.2.1. Participants

**Figure 1:**
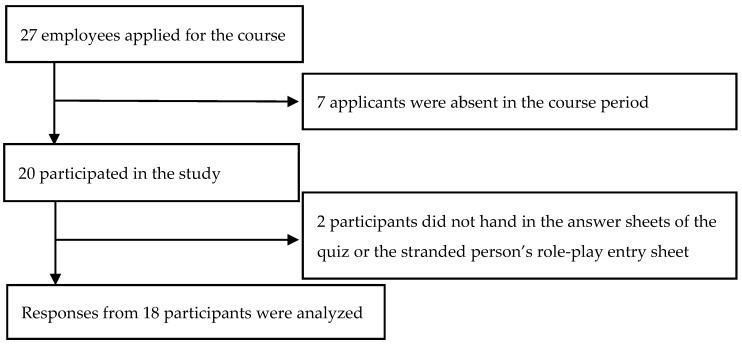
Tree diagram of the change of the sample.

The participants were influential employees in the firms, including director-class personnel and those who engaged in the BC promotion. We set the recruitment goal at 40 applicants and received 27 applications, but seven applicants were absent in the course period; thus, we had a total of 20 participants in our study (19 men and one woman) (Figure 1).

Since the course required someone to play the unique role of stranded individuals, including those who needed special assistance, some of the authors (Y.S., N.S., S.B., and K.S.) took part in the role-play as four stranded persons, including vulnerable people, and three males from the model facility (a commercial complex) took part as staff members in charge of receiving stranded individuals. We did not include these seven course organizers as participants in the analysis.

#### 2.2.2. Recruitment Method

Staff members of Tokyu Facility Service Co., Ltd. held briefings regarding the “BC training program”, including our course, for several days from 25 March 2019 at individual offices of 20 companies that cover a wide range of industries dealing with transportation, real estate, and lifestyle-related services, and collected e-mail applications for the basic course. During the period from mid-June to 8 July 2019, we accepted applications for our course, also by e-mail.

### 2.3. The Outline of the “Role-Play” in the Course

#### 2.3.1. Role-Play Setting

We set the role-play condition as the firm receiving stranded people in the event of the Tokyo metropolitan earthquake. We simulated the situation where all lifelines, including electricity, gas, and water, had stopped, and participants experienced the life of disaster victims. They endured meals limited to emergency rations that did not require cooking, using the emergency toilet which needed to be covered with a defecation bag and a coagulant applied to tie its mouth for disposal, and sleeping in the bedding material stocked in the facility. Since we assumed that the course was conducted in the model facility, we took the training materials from their stockpile, including emergency rations and schlaf sheets for bedding. The course was held in August, and we prohibited the use of air conditioning from 18:00 to 19:00 h on the first day to simulate the mid-summer conditions without electricity.

#### 2.3.2. Events in the Role-Play

In the role-play, we set a series of conceivable events in receiving vulnerable people. Table 2 shows the contents and learning goals of the primary events set for dealing with vulnerable people.

We included the role of a “local notable with platinum card membership who was also a valued customer for the shelter-offering business enterprise” (hereinafter referred to as the “local notable with platinum card membership”) within that of the vulnerable people, in the context of BC’s significance regarding the care provided to important customers and five types of vulnerable people, such as lactating woman and elderly people. A total of 20 participants were divided into two groups, four facility staff members (hereinafter referred to as “facility members”) and 16 stranded people. As mentioned earlier, we added three facility members from the model facility and four stranded people, including the authors playing vulnerable people; we had a total of seven facility members and 20 stranded people in the role-play. Facility members set up the shelter space, including the zoning, resembling the actual disaster event. Since the stranded would have to wait outside the facility until the shelter space is ready to accept them, we instructed those assigned to this role to wait in another room. When the facility was almost ready to start the reception, we instructed them to wait outside. The members of Tokyu Facility Service Co., Ltd. randomly named four people to the roles of vulnerable people (the roles of an Indonesian couple, a lower grade elementary school boy with wheat allergy, and a local notable with platinum card membership) from those who were assigned to the roles of disaster victims, so that the casting was unbiased regarding the participants’ business affiliations. We held a briefing to explain their roles while they waited and asked them to perform the event’s scene in the role-play. As for the roles of the Indonesian couple, we named only female participant for the wife’s role, and a male participant who could speak English for the husband’s role. Three of us took the roles of other women who were stranded (the roles of a lactating woman, an older woman with dementia, and a young woman). After the role-play, we held a meeting with discussion and commentary to review proper assistance for and the characteristics of vulnerable people.

### 2.4. Evaluation Method

#### 2.4.1. Quiz on Vulnerable People

We organized a quiz for the participants regarding the events in the role-play, on the following three items: “(1) Halal foods (select foods and dishes prohibited to Muslims from 16 candidates)”; “(2) Food allergies (identify allergic ingredients of wheat, buckwheat, egg, milk, and peanut contained in the given six types of foods and dishes often served in disaster events)”; and “(3) Meals for older people (select hard to chew and swallow foods and dishes for the elderly people)”. The quiz was implemented once before the role-play; we implemented the same quiz again after the discussion and commentary about the events of the role-play.

#### 2.4.2. Stranded Person’s Role-Play Entry Sheet

We provided the participants with entry sheets with free columns next to event descriptions so that they could enter what they had learned in the role-play.

#### 2.4.3. Post-Course Questionnaire Survey

##### Self-Evaluation of Facility Members

After the course, we asked the four participants, who played the role of facility members, to self-evaluate their performance in dealing with vulnerable people. We asked them to evaluate either with “yes” or “no” whether they could provide them with proper support.

##### Evaluation of Emergency Rations

We asked all participants to evaluate the emergency rations served in the course, wherein curry rice with a flameless ration heater was served as the first-night dinner, canned bread (two pieces) at the second-day breakfast, and ready-to-eat porched gomoku-gohan (rice cooked with several ingredients like carrots, mushrooms or chicken) at the second-day lunch. We asked them to evaluate meals by rating each either with “fine” or “requires improvement,” and those who answered with the latter were requested to freely describe the specific points of improvement.

##### “Learned for the First Time,” “What Left an Impression,” and “What Made to Think about.”

We asked the participants to comment freely on the following three categories, regarding their experience during the course: what they learned for the first time, what left an impression on them, and what made them think. We categorized the answers into groups based on content.

### 2.5. Data Analysis

We used the Wilcoxon signed-rank test at the 5% significance level to compare the score of the quiz implemented before and after the course, regarding dealing with vulnerable people. We used IBM SPSS statistics Version 20 (IBM Japan, Ltd.) for statistics analysis.

As for the free comments, we used a KJ method [17] software (ISOP Chohassouhou Ultra Presen Version 4.0.0 (ITEC, Tokyo, Japan)) to organize opinions from the participants into a data card of a summarized, short paragraph. When a participant provided several opinions, we summarized the contents and allocated one data card per opinion. We also organized data cards with similar content into groups and named each group.

### 2.6. Ethical Considerations

This study was conducted under the approval of the ethics committee of the Human and Social Sciences Department, Ochanomizu University (Notification No. 2019-11). All participants were briefed and explained in writing in advance that they were going to be asked to enter their names in all answer sheets provided in the course, such as the quizzes about vulnerable people or the stranded person’s role-play entry sheet, but it was up to them if they would allow us to use the study results and that they would suffer no disadvantages if they decided to prohibit us from using the data. We received consent from all participants on the first day of the study.

## 3. Results

We analyzed data provided by 18 participants, except for the two who did not hand in the answer sheets of the quiz or the stranded person’s role-play entry sheet. Not all items were answered, and we deleted unanswered items; the number of valid responses differed in items.

### 3.1. Scores of the Quizzes about Vulnerable People

Table 3 shows the score results of the quiz, which indicates a significant score increase in all three categories of the quiz after the course.

### 3.2. Stranded Person’s Role-Play Entry Sheet

We asked participants to comment freely in the entry sheet what they would do if they were in the same situation, as described in the primary events shown in Table 2. Table 4 shows the results of the collected answers.

Some commented that they need to “improve the stockpiles” to satisfy the requests for Halal foods or baby foods for future improvement, while others showed a positive attitude to “investigate possible substitutes” for items requested by vulnerable people. As for a few sensitive items, such as the care of those with platinum card memberships or the considerations for women’s privacy, some answered that they would try and persuade them to tolerate the inconvenience.

### 3.3. Post-Course Questionnaire Survey

Table 5 shows the results of the self-evaluation of facility members who received stranded persons.

There were some items that no one successfully responded to, such as dealing with Halal foods or allergic foods. We also learned that vulnerable people were served only by specific individuals among the facility members.

As for the evaluation of the emergency rations provided by the model facility (n = 17), ten participants (58.8%) answered “fine as they are” and seven (41.2%) answered, “require improvement”. The improvement suggested most was to “improve the variety of dishes to satisfy the special needs based on religious or health grounds”. Remarks on the food taste like “The gomoku-gohan tasted bad” were also observed.

Regarding free comments about “Learned for the first time”, “What left an impression”, and “What made to think about”, we sorted the collected comments and organized those relevant to serve the two purposes of this study, namely, “Regarding vulnerable people” and “Regarding the lives of those affected by disasters” into Table 6.

Comments about the condition of the accommodation were the most observed ones.

## 4. Discussion

### 4.1. Understanding about Vulnerable People

This is the first paper that deals with stranded persons and their life in a temporary shelter. Unlike affected residents who are accommodated in their municipal shelters, stranded persons are often taken care of by a business establishment at their destination. Children, the elderly, women, and patients are widely known as vulnerable people in disasters and refugee camps. In addition to them, important customers are also regarded as a target for special care in a shelter operated by a company. This study has a different perspective from other research on shelter management. Unique consideration and investment are required in taking in stranded persons for profit over the long run.

#### 4.1.1. Quiz Score Improvements

The quiz score showed a significant improvement in the comparison between before and after the course (Table 3); we confirmed that the course contributed to improving the correctness of knowledge. We also confirmed many comments regarding vulnerable people, in the “Remarks” entry (Table 6), which suggests that the course had a considerable learning effect surrounding vulnerable people.

A study has reported that many of the disaster victims with foreign nationalities named “meals” to be the most critical issue in past earthquakes, for they did not serve Halal or vegetarian meals in the temporary shelters at that time [18]. In this era of globalization, tourists are frequently caught up in crisis events, such as natural disasters, and outbreaks of diseases, such as pandemic influenza and COVID-19. In spite of the vulnerability of international visitors, who are away from their home area and have limited local knowledge, research, outside the tourism field, has not generally identified this particular group of people as a major target group for emergency planning [19]. Japan enacted the Basic Act for Promoting Tourism-Oriented Country in 2006 and the security of inbound tourists is an issue of vital importance. Many other countries that financially depend on tourism also need to prepare for emergencies. Given the recent frequency and intensity of crises around the globe, certified crisis-prepared destinations may have a market advantage [20]. Appropriate care for customers could increase a business’s credibility because of the public commitment to quality, safety, and risk management with accordant rise in sales, competitiveness, and profitability [21].

Another study reported cases wherein the victims had a difficult time with meals because the shelter management had little understanding of food allergies [22]. As for some older people having difficulty in chewing/swallowing, rice balls or bread provided in disaster events is not suitable [23]. Considering that over 27% of the Japanese population is now older than 65 years, it should be highly probable that the business enterprises will receive stranded elderly people in disaster events. The current issue requiring the utmost urgency is the lack of sufficient stock space [24]; thus, food stockpiles should be prepared according to the conditions of the weakest victims to make the most of it because the emergency rations targeting them are also applicable for healthy people [25]. It should be mandatory for the firm’s risk management to prepare and prevent food-related health hazards, including direct ones like anaphylaxis shock due to allergic foods or secondary hazards like aspiration pneumonitis among older people [26]. Japan’s population aging rate (the percentage of people aged 65 years and over) exceeded 7% in 1970 and 14% in 1994 [27]. Japan’s speed of population aging is the fastest in the world. At some future date, other countries will experience similar situations to that which Japan faces now. In particular, emerging nations in Asia could learn a lot from advanced efforts made by Japan since eight cities of the top ten highest risk of earthquake are in Asia [28].

#### 4.1.2. Remaining Issues

There were some opinions among participants about the local notable with platinum card membership that they “would not treat them with special care,” or about woman’s privacy issues that they “would persuade her to tolerate inconvenience”. These opinions suggest the lack of understanding of customer service in disaster events. If a business enterprise fails in treating the local notable and the customer with platinum card membership, it will have significant impacts on its BC. Because it is highly possible that they are important customers for the business enterprise. As opposed to the shelters managed by the municipal governments that value fairness in treating disaster victims, the business enterprise probably will be expected to treat such customers with the utmost care.

As for the gender-specific needs of women, a study reported that the lack of sufficient understanding and support from the shelter management caused problems in the past disasters like the Hanshin Awaji Earthquake in 1995 [29], suggesting that the issue requires further improvement. In the Great East Japan Earthquake in 2011, most of the members overseeing shelter management were male; gender-specific requests or needs were hardly reflected [29]. Since the members overseeing the stockpile delivery/inventory were also male, it was quite embarrassing for female victims to ask them for sanitary items [30]. Sanitary items such as disposable pads have built confidence around preventing leakage and increased their ability to engage in daily activities of living [31], so it is important to stock the sanitary items in the disaster events. The shelter management guidelines issued by the Cabinet Office instruct the appointment of female members in the shelter management committee so that it is operated by including a female standpoint [32]. Female facility members are also required to receive the victims to satisfy the gender-specific needs regarding stockpiles or privacy considerations. Among the necessary care for female victims, proper consideration and assistance should be mandatory in care for the pregnant or lactating women because it is directly connected to the well-being of the unborn child or infants and their mothers. A study reported that others got disturbed while sleeping when infants cried due to milk shortages in the Great East Japan Earthquake [33]; this suggests that the shelter environment supporting vulnerable people should also be comfortable for other victims.

#### 4.1.3. Workload of Employees

There were several items wherein the facility members answered: “Do not take action” (Table 5), showing a significant difference among facility members regarding the number of times they assisted or contents of the assistance they provided for those who required it. Although participants have opportunities to review the role-play events in the course to follow up on the events where they could not take proper actions, only those who take action in the role-play can learn through experience; further improvement in course planning should be necessary for the future. In the disaster events, they should avoid the situation wherein the workload of receiving stranded people concentrates on a specific group of employees from the BC standpoint. Another study indicated that the municipal government officials engaged in disaster recovery efforts like shelter management could suffer from prolonged stress-induced symptoms by being overwhelmed by the mental and physical pressures [34]. In this context, it is also mandatory for the business enterprise to consider the well-being of its employees engaged in receiving stranded people, whose responsibility could be prolonged due to the progress of the recovery efforts. Formulating a proper workforce plan with balanced task-sharing among employees should be necessary. As for the event setting in the role-play, wherein the local notable with platinum card membership raises his voice to complain about the crying baby, there was a comment in the entry sheets (Table 4) “to respond with multiple members of staff,” which is a useful measure in handling complaints in ordinary situations.

### 4.2. Commodities Necessary in the Life of Disaster Victims

#### 4.2.1. Evaluation of Emergency Rations

Among the comments of “requiring improvement” in the evaluation on the emergency rations, there were comments regarding the taste of the food like “gomoku-gohan tasted bad,” and to “improve food variety to satisfy religious or health requirements of the victim.” Unlike normal circumstances, the variety of the meals would be quite limited in the disaster events, and the business enterprise should improve its food stockpile with dishes at an acceptable level of quality [35]. A hands-on program like the course we implemented in this study should serve the business enterprise promoting BC measures in receiving stranded people to improve its food stockpile by learning from valuable comments like “did not taste good” through tasting in the simulation training. Satisfying physiological needs such as meals should enable the business enterprise to practice the Business Continuity Plan (BCP) in the disaster events [36]; emergency rations are indispensable for the stranded people and the employees taking care of them. According to the level of severity, one may need to endure the life of disaster victims for quite some time; a business enterprise unable to secure a sufficient level of food supply for its employees engaged in BC practice would not live up to the BCP [35]. The business enterprise must prepare and secure proper stockpiles, including emergency rations, as a part of its BC promotion and preparedness against future disasters.

#### 4.2.2. Bedding Apparatus Stocked in the Facility

Through the simulation of the sleeping condition, we learned that the participants experienced difficulties in sleeping from noises like the snoring of others, as well as bodily discomfort from sleeping on the floor only with schlaf sheets. In the sleeping condition simulation, the previous study demonstrated the importance of the mattress [24]; it is essential in the mentally and physically stressful life of the disaster victims to secure bedding apparatus that is comfortable enough to ensure sound sleep. Even in the short training course of a two-day one-night period subjecting healthy participants, as we did in our study or in the previous study, the participants complained about discomfort in sleeping on the floor [24]. According to a study, the elderly victims in past earthquakes suffered from a performance decrease in activities of daily living (ADL) caused by sleeping directly on the floor [33]. This should be a more pressing issue for vulnerable people. A possible solution for the problem would be the use of the cardboard bed: it is useful for preventing the severe decrease in ADL capability of elderly victims and for preventing respiratory diseases by providing users with a certain level of height from the floor, as one could inhale dust by sleeping directly on the floor [37]. As shown here, a cardboard bed has various merits, including the prevention of secondary health hazards during the shelter life or securing personal space for each victim [24]. Using only the bedding apparatus stocked in the model facility in the simulation of the disaster victims’ shelter life provided the opportunity to discover essential items in a sheltered life through experiencing mental and bodily stress; it was a valuable opportunity to reconsider the stock of bedding apparatus for the future disaster events.

#### 4.2.3. Crisis Preparedness and Business Continuity

Although stockpiling the emergency rations and bedding apparatus takes money and stock space, a significant positive impact of organizational crisis preparedness on the business success has been confirmed [38]. For private industries, there are two risk management choices of insurance and investments in self-protection to reduce expected losses due to disasters [39,40]. While direct disaster losses can be measured and insured, bad reputation about the poor treatment of affected customers during disasters cannot be compensated later. Unlike a physical damage to a building, health complaints among employees need to be addressed immediately for BC. To maintain health conditions, improved stocks of food and bedding are essential and government subsidies have the potential to encourage them [41].

### 4.3. The Limitations of Our Study

Since the course was implemented as an optional program in the BC training program all participants had a rather high level of BC awareness, which could have enhanced the overall training effect compared with the results in training of the public. Moreover, data analysis was conducted for 18 participants in total, with only one female, which limits the generalization of the results. We should improve the training methods, such as implementing different approaches according to the actual conditions of each participant, to continue human resource fostering programs, like the course in this study. Besides, seven participants canceled at the last minute, probably because all of them were director-class personnel who often need to deal with sudden situations. Since the director-class personnel manage the BCP practice [42], receiving them, although small in number, created an impact from the standpoint of promoting support measures for stranded people in the firm.

## 5. Conclusions

We implemented two-day and one-night human resource fostering programs for promoting support measures for business enterprises to deal with stranded people. The first goal of our study was to improve the knowledge and understanding of participants about vulnerable people. This was demonstrated by the analysis results, such as the significant increase in scores in quizzes conducted before and after the course lectures on customer relations focused on such victims during disaster events, by learning through a series of role-play and review sessions. The second goal of our study was to reconfirm the necessary commodities for the shelter life of limited lifeline and supplies. They were discovered by meal or sleep training using only items stocked in the model facility to evaluate emergency rations or experiencing discomfort from sleeping on the floor, from the perspectives of disaster victims and vulnerable people. The training should be implemented regularly in the future to ensure a smooth reception of stranded people during disasters.

## Figures and Tables

**Table 1:** Organization of the course.

Time	Course Details	Purpose
15:00~15:30	Reception	—
15:30~16:30	Lecture about business continuity and disaster food supplies	Learning how disaster foods are essential to business continuity for companies
16:30~16:45	Break	—
16:45~16:50	Answers and submission of quiz regarding vulnerable people	Ascertaining participants’ level of knowledge about vulnerable people before the role-play activities
16:50~17:10	Orientation and submission of consent forms	Explanation of the role-play activities
17:10—Next morning	Role-play (facility staff members and stranded persons) Filling in the stranded person’s role-play entry sheet while watching role-play Meal practice using actual emergency ration (first day dinner and second day breakfast) and accommodation practice	Experience of providing response to stranded persons and of receiving aid Review of stockpiles by using them (meals, toilets, and bedding) in the same way as during an actual disaster
8:30~9:00	Sharing of impressions on performance of the roles of vulnerable people (all the seven people) and facility staff members (selected one person)	Participants other than the person presenting share what they learned by playing their roles
9:00~10:00	Role-play review and explanation	Lecture on correct responses to events during role-play
10:00~10:15	Break	—
10:15~11:00	Group discussion (1) and presentations	Regarding responses to customers during disasters, a debate centered on the response to vulnerable people will be held
11:00~12:15	Group discussion (2) and presentations	Discussion of hypothetical issues in case taking in stranded persons continues for four days after the disaster and one week after the disaster
12:15~13:00	Meal practice (lunch) Second test regarding vulnerable people Filling in follow-up survey Submission of entry sheet (stranded persons only) and follow-up survey	Through role-play and review discussions, an evaluation will be made as to whether participants obtained knowledge about vulnerable people regarding the same topic The follow-up survey analyzes self-evaluations by facility staff members regarding vulnerable people during role-play activities, evaluations of emergency rations in a model facility by participants, and overall impressions of the course

Practice relating to the limited use of water and sewer services and lighting and the use of toilets for disasters was held between 17:10 on the first day and 13:00 on the second day, and training on the prohibited use of air conditioning was held from 18:00~19:00 on the first day.

**Table 2:** Details of the main events during the role-play related to seven vulnerable people.

Vulnerable People (Number)	Event Time Slot	Event Details	Response to the Event and Learning Aims after Review/Explanation
Indonesian couple (2 people) nominated from among participants	First day dinner practice	Request for Halal food	Learning about foods that cannot be eaten by Muslims and about stranded persons who require special consideration regarding their meals because of religious reasons
Lower grade elementary school boy with wheat allergy (1 person) nominated from among participants	First day dinner practice	Sudden vomiting after eating curry	Learning about the precautions to be taken when dealing with vomit and about using the treatment kit
	After first day dinner—accommodation preparation	Vomiting after receiving a Bisco (a cream sand biscuit) from an old lady with dementia	Awareness of the need to understand the cause for vomiting
		A young woman who noticed that the child has an allergy badge conveys this to the facility staff	Learning about allergy badges and understanding the need to share information about allergy sufferers with nearby stranded persons and facility staff members
	Second day breakfast practice	Vomiting after touching breadcrumbs on the floor	Learning how symptoms can occur not only by consuming allergy-inducing food but also by contact or absorption
Lactating woman (1 person) Second author	First day dinner practice	Request for baby food	Learning about the need for stockpiles of baby food and about suitable baby food as per babies’ age
	After dinner on the first day—accommodation preparation	There is a need for a place to nurse a baby, but the boy who vomited is sleeping in the tent *^1^, so it cannot be used	Learning about the importance of support to enable lactating woman to continue breastfeeding during disasters, including providing a quiet location for breastfeeding and having consideration for their privacy, etc.
		Request for baby milk formula and a baby bottle; although the mother tried to give the baby breast-milk before, it was not possible because of the unfamiliar environment	Becoming aware of the need for stockpiles of baby milk formula and baby bottles and learning to use liquid baby milk formulas, the ban on which was lifted in Japan in August 2018
	While going to bed on the first day *^2^	A nine-month-old baby does not stop crying because its diaper is soiled. A request is made for a new diaper	Learning about the need for stockpiles for necessary items such as diapers and waste bins and about the sizes of diapers according to children’s developmental stage
Young woman (1 person) Sixth author	First day After dinner—accommodation preparation	After learning how to use the emergency toilets, a young woman complains that she would feel uncomfortable being seen by other people while carrying used defecation bags and throwing them into waste bins in the corridor	Becoming aware of the need for considerations for privacy in facility management
		Request for sanitary products	Learning about the need for women-specific emergency stores
		A young woman complains that she does not want to sleep in the same room as men	Learning about gender issues and the danger of sexual crimes during disasters
Older woman with dementia (1 person) First author	Second day breakfast practice	Difficulty in opening a tin of bread; a woman tells the facility staff that the older woman is struggling after choking on the dry bread that she was eating	Learning about meals that are difficult for older people to eat and about the danger of aspiration pneumonia
		A young woman tells the facility staff that she saw in a TV program that “putting bread in a plastic bag and soak it in hot water makes it easier to eat”	
Local notable with platinum card membership (1 person) nominated from among participants	First day dinner practice	Complains about the boil-in-the-bag curry; requests for side dishes to be brought from a store	As relationships with clients that are important to the company are also important during disasters, understanding the importance of responding correctly to requests during disasters
	After dinner on the first day—accommodation preparation	Requests to be able to use the only bunk bed	Learning about responding to demands from clients who are important to the company and about the benefits of bunk beds
	While going to bed on first day *^2^	A nine-month-old baby does not stop crying because its diaper is soiled. The person shouts at the baby for being noisy	Learning about responding to difficulties between important clients and nearby stranded persons

*^1^ There was an individual-size tent in a shelter space; *^2^ The actual time of going to sleep was after the completion of the role-play activities.

**Table 3:** Change in knowledge regarding vulnerable people (n = 18).

Test Items (Max. Score)	Mean Score ± SD		Wilcoxon Signed-Rank Test
	Pre-Course	Post-Course	
Halal food (max. score 16.0)	12.2 ± 1.26	14.2 ± 2.44	*p* = 0.005
Food allergies (max. score 30.0 *)	22.8 ± 4.51	26.7 ± 2.22	*p* = 0.002
Meals of older people (max. score 6.0)	3.06 ± 1.51	5.89 ± 0.47	*p* < 0.001

* Regarding all six types of foods, the maximum score was 6 × 5 = 30.0 because of the format of selecting five types of food-based allergies: wheat, buckwheat, egg, milk, and peanut.

**Table 4:** Lessons learned and impressions after seeing events regarding vulnerable people and how one could respond personally (n = 13).

Event	Lessons Learned and Impressions after Seeing Events Regarding Vulnerable People and How One Could Respond Personally (Answered Directly after Seeing the Event) *^1^
Response to an Indonesian couple who require Halal food	Provide a polite explanation if there is no Halal food (5) *^2^
	Think in advance about providing for Halal food (4)
	Ask them to manage with only rice (2)
	Provide maximum support because it is part of the national character
	Performance impressions: Incorrect advice was given (bread containing animal fats, which are forbidden to those who require Halal food, and mixed rice containing sake were offered)
Response to a local notable with platinum card membership who demanded side dishes from the store instead of curry rice with flameless ration heater	Not treat them with special care (8)
	Make arrangements with tenants in advance
	If side dishes are unavailable, present him with alternatives to choose from
Response to a lactating woman who requires baby food	Keep baby food in stockpiles (4)
	Look for alternative foods (2)
	If there is none, give a polite explanation (2)
	The facility staff gave a polite response
	Check if there is no baby food
Response to vomiting by lower grade elementary school boy with wheat allergy who ate curry	Respond using a waste disposal kit (3)
	Check what the child ate and give him/her water (3)
	Move nearby people to another location and deal with the waste (2)
	Isolate the child (2)
	Cooperation is needed from everyone, not just the facility staff members (2)
Response to a young woman who expresses discomfort in being seen dealing with excrement	Response should be provided by female staff (if there are none, ask for help from stranded persons) (3)
	Explain that it is an emergency and fortitude is needed (3)
Response to vomiting by lower grade elementary school boy with wheat allergy who ate Bisco	Let the (child) rest (3)
	Move the child away from older woman with dementia and take away the Bisco (3)
	Staff require masks and gloves and should carefully disinfect the area (2)
	Isolate the child to curb the possibility of infection
	Becomes an issue if the response is slow
	No medical care for the child
Response to a young woman in need of sanitary products	Stockpiles are required, as with diapers (6)
	Use a towel instead if there are no sanitary products
	Look for a female volunteer
	Female staff are required
	Make arrangements
Response to a lactating woman who requires a location for breast-feeding	Use a substitute location (e.g., partition using cardboard) (7)
	Ask for help from other women (2)
	Ask the individual for her ideas
Response when someone has an allergy badge	Communicate with the staff Find out what the allergy is if possible (5)
	Ask the child (4)
	It is necessary to get a better understanding and knowledge of any help marks or maternity mark, etc. in advance
	Is it possible to deal with the allergy?
Response to a lactating woman who requires a baby bottle for a nursing child	Required in stockpiles (6)
	Environment for sterilizing baby bottles is also required
	If there are none, use coffee creamer, and plastic bottles
	Make arrangements
Response to a local notable with platinum card membership who requests a makeshift bed	Do not treat him with special care (11)
Response to a young woman who is not comfortable sleeping in the same room as men	Make a women-only section, or take her to an area where there are many women (10)
	Persuade her (2)
	Request cooperation from those in the location
Response to a lactating woman who requires a replacement diaper	Emergency stores are required (3)
	Replace with towel (3)
Response to a local notable with platinum card membership who shouts at crying infants	Persuade him (6)
	The response from a single member of staff is limited, so it is better to respond with multiple members of staff
Response to an older woman with dementia who cannot eat bread	Respond with support such as providing water to drink (3)
	Open the stiff lid, and give it to her
	Give her other food
Responding to advice from a young woman about the diets of older woman with dementia	Try the suggestion (4)
	Be cautious about unconfirmed information (3)
	Preparations are required in advance
Responding to lower grade elementary school boy with wheat allergy who is vomiting after touching breadcrumbs on the floor	Cleaning is required (3)
	Get cooperation from those nearby (3)
	Isolate the child (2)
	Take the child to hospital

*^1^ Indecipherable or incoherent responses were excluded. *^2^ The numbers in parentheses indicate the number of answers with the same content.

**Table 5:** Self-evaluation about response to vulnerable people by those in acceptance roles (n = 4, facility staff members shown as A, B, C, and D).

		Do you Think You Could Provide Proper Support?		
Items for Consideration	Evaluation Items	Yes	No	Do Not Take Action
Response regarding Halal food	Knowledge of Halal food	-	B, D	A, C
	Preparation of disaster food in response to Halal food	-	B, D	A, C
	Response when told that the provided emergency ration cannot be eaten	B	D	A, C
Response to food allergies	Ascertain the existence of food allergies	A	B, C, D	-
	Preparation of allergy response foods	C	A, B, D	-
	Response when allergy symptoms occur Response to the person feeling unwell and processing of vomit, etc.	A, B, C, D	-	-
	Knowledge of food-based allergies	-	A, B, C, D	-
	Information sharing when there is a food allergy sufferer	-	A, B, C, D	-
Response to lactating woman	Response regarding baby food	B, C	D	A
	Assignment of rooms separated from other stranded persons	A, B, C, D	-	-
	Preparation of nursing room	A, C, D	B	-
	Response regarding milk for infants	B, C, D	-	A
	Response regarding a nursing child that does not stop crying	-	B, D	A, C
	Response regarding diapers	-	B, D	A, C
Response to young woman	Consideration for privacy	B, D	-	A, C
	Response regarding sanitary products	-	B,D	A,C
	Gender-based zoning	A, D	B	C
Response to older woman with dementia	Response at the time of reception	B, D	-	A, C
	Explanation of how to use facilities and stockpiles	B, D	-	A, C
	Response regarding setting aside food, considering that she is unable to eat a large amount at one time (from a standpoint of prevention of food poisoning)	B, D	-	A, C
	Response after dirtying the toilet	B, D	-	A, C
	Response regarding dealing with other stranded persons	B	D	A, C
	Considerations for meals (Unable to open lids and prone to choking)	A, B, D	-	C
Response to local notable with platinum card membership	Request for special treatment	A, B, C, D	-	-
	Complaints about other stranded persons	D	B	A, C

**Table 6:** Answers regarding vulnerable people and those whose lives are affected by a disaster after taking this course from among “Learned for the first time”, “What left an impression”, and “What made to think about” (n = 18).

Group Name Compiled by KJ Method *^1^	Questions from Post-Survey	Free Comment *^2^
Regarding vulnerable people	Learned for the first time	Response to Halal food and allergies (2)
		Specific details of Halal food
		Response to vulnerable people is not “irregular”
		The necessity for preparations for vulnerable people (physical items)
		The term “CHECTP” *^3^
		Oral uncleanness can lead to illness (in older people) and that older people may experience difficulties in drinking water
	What left an impression	Difficulty and importance of responses regarding CHECTP
		The number of vulnerable people is much more than expected!
	What made to think about	It is necessary to consider how to respond to vulnerable people during normal times
		The danger of mistakenly responding to ailments that cannot be seen such as allergies among children
		Help is needed for the response regarding vulnerable people
		It is necessary to acquire knowledge about responses regarding vulnerable people such as those with allergies
Regarding the lives of those affected by disasters	Learned for the first time	Gain a new understanding of the various issues by experiencing being a victim
		The snoring of other people when sleeping was more bothersome than previously thought
		The importance of securing necessary personal space during a disaster
		It is necessary to sign the explanatory documents to receive people
		The method of eating disaster food
		The difficulties experienced during power blackout/water outage situations, including toilet practice
	What left an impression	The air conditioning was strong, and I was cold when sleeping (2)
		Snoring at night was surprisingly bothersome (including my own snoring)
		It made me realize anew that “inconveniences” can make us vulnerable
		Sleeping in a sleeping bag on the floor is tough on the body
	What made to think about	If there is no air conditioning, the only thing to do at night is to go to sleep early
		It is necessary to have space to change clothes
		I was a bit concerned about how long this situation would continue
		How I should act in my position if I became a victim while at work or at home
		I have never experienced being a victim, so there were many things I thought would be easier

*^1^ Grouping of similarities in free comments after participants were asked to respond with expressions such as “Learned for the first time”, “What left an impression”, and “What made to think about”, after taking the course. *^2^ The numbers in parentheses indicate the number of answers with the same content. *^3^ In the lecture on role-play review and explanation, vulnerable people during disasters are shown by the initials C: child, H: handicapped or persons with disability, E: elderly people, C: chronically ill, T: tourist, and P: pregnant woman.

## Appendix A. Quiz on Vulnerable People

**Table A1:** **Q1.** Please circle the items that cannot be eaten by those who require Halal food (please circle all that apply).

1. rice	2. wheat	3. beef	4. pork
5. chicken	6. fish	7. egg	8. alcohol
9. retort-pouch curry	10. deep-fried food in boxed lunch/carry-out	11. sugar	12. salt
13. vinegar	14. soy sauce	15. miso	16. sweet sake

**Table A2:** **Q2.** Please circle allergic ingredients in each dish (please circle all that apply).

Dish	Ingredients that Cause Food Allergy				
1. beef stew	wheat	buckwheat	egg	milk	peanut
2. pork curry	wheat	buckwheat	egg	milk	peanut
3. hamburger steak	wheat	buckwheat	egg	milk	peanut
4. sweet bread	wheat	buckwheat	egg	milk	peanut
5. tomato and cheese pizza	wheat	buckwheat	egg	milk	peanut
6. crab cream croquette	wheat	buckwheat	egg	milk	peanut

**Table A3:** **Q3.** Please circle the items that are difficult to chew/swallow for the elderly (please circle all that apply).

1. cold rice ball	2. bread
3. cold boxed lunch	4. miso soup
5. canned fish	6. jelly drink

## References

[1] Uchiyama S., Yanagida Y., Murakami M., Mizuno O. The Urban-Area Mitigation System for Providing Information and its Generalization. Proceedings of the 33rd International Conference on Information Networking.

[2] Cabinet Office (Disaster Management) (2015). Guidelines for Measures to Aid Stranded Persons in the Occurrence of a Large-Scale Earthquake.

[3] Shirota Y., Hashimoto T., Sakura T. (2012). A causal analysis on stock price appreciation of certain firms after the Great East Japan Earthquake by an approach of web text-mining. J. Dep. Econ. Gakushuin Univ..

[4] Nakayachi K., Kudo D., Ozaki T. (2014). Trust in organizations concerned with risks of the Great East Japan Earthquake. Jpn. J. Psychol..

[5] Mori S. (2012). Traffic management on regional disaster prevention plan about sufferers cannot take homeway. Kōtsūken.

[6] Yang Z., Inagaki K., Yoshida S., Sadohara S. (2015). Regional characteristics analysis from the view of community-based disaster risk management for people with functional needs in times of disaster. J. Soc. Saf. Sci..

[7] Ito Y., Asama Y. (2015). Foreign evacuee and disaster multicultural coexistence. Bull. Support Cent. Revival Educ. Miyagi Univ. Educ..

[8] Adachi Y., Katsunuma T. (2011). Response to and issues faced by asthmatic and allergic children during disasters: Report and proposals for the future based on the experience of the Great East Japan Earthquake 5. Response pamphlet (1) Introduction. Jpn. J. Pediatric Allergy Clin. Immunol..

[9] Kataoka K., Sato S. (2019). Case report of disaster response training in private companies: Application of incident command system in provide companies. J. Natl. Inst. Public Health.

[10] Oizumi S., Kikuta K., Hayama H., Mori T. (2013). Actual utilization of heating and measures against cold on elementary school gymnasiums in cold and snowy regions part 3 measurement results of evacuation training and energy consumption characteristics of gymnasium-contained elementary school. Tech. Pap. Annu. Meet. Soc. Heat. Air-Cond. Sanit. Eng. Jpn..

[11] Suzuki Y., Une H., Kubo J. (2017). What we can learn from the Kumamoto Earthquake—Transmissions from geography. E-J. GEO.

[12] Hyakuta T., Nakanobu R. (2011). Effect and future task of “the practice for the simulated shelter experience”. Bull. Jpn. Red Cross Hiroshima Coll. Nurs..

[13] Nishigami A., Zhang X., Miura A., Iwasa M. (2018). The report on overnight stays at simulated shelters for disaster nursing practicum. Baika Women’s Univ. Res. Bull. Fac. Nurs. Health Care.

[14] Lee Y.-I., Trim P., Upton J., Upton D. (2009). Large emergency-response exercises: Qualitative characteristics-a survey. Simul. Gaming.

[15] Yoshida K., Kitamura T., Izumi T., Nakatani Y. A simulation system of experience with a disaster by locating memories on a virtual space. Lecture Notes in Computer Science, Proceedings of the 18th International Conference on Human-Computer Interaction, HCI International, Toronto, ON, Canada, 17–22 July 2016.

[16] Alexander D. (2000). Scenario methodology for teaching principles of emergency management. Disaster Prev. Manag. Int. J..

[17] Scupin R. (1997). The KJ method: A technique for analyzing data derived from Japanese ethnology. Hum. Organ..

[18] Abe M. (2017). Shelter management and response to foreign evacuees as seen during the Kumamoto Earthquake. Disaster Recovery Revital. Rev..

[19] Morakabati Y., Page S.J., Fletcher J. (2017). Emergency management and tourism stakeholder responses to crises: A global survey. J. Travel Res..

[20] Pennington-Gray L., Schroeder A., Wu B., Donohoe H., Cahyanto I. (2014). Travelers’ perceptions of crisis preparedness certification in the United States. J. Travel Res..

[21] Conroy M. (2001). Can advocacy-led certification systems transform global corporate practices?. Evidence, and some theory. Work. Pap. Ser..

[22] Yamaoka A., Abe H., Watanabe Y., Kakuta H., Umebayashi H., Inagaki T., Abukawa D., Yanagida N., Minoura T., Morikawa M. (2011). The questionnaire for caregivers of allergic children in the Great East Japan Earthquake. Jpn. J. Pediatric Allergy Clin. Immunol..

[23] Beppu S., Aoyama K. (2008). A study on the affected livelihood and its problems due to Niigata Chuetsu Oki earthquake. Annu. Rep. Res. Cent. Nat. Hazard Disaster Recovery Niigata Univ..

[24] Sudo N., Mashiro G., Beppu S., Hakamata R. (2019). A training program to enhance disaster preparedness of group companies in the Tokyo metropolitan area. Int. J. Environ. Res. Public Health.

[25] Ito A., Mineo S., Watanabe T., Beppu S., Abe N., Kawai Y., Yoshida Y. (2017). The institution of disaster food certification system for people with dietary problems. J. Jpn. Disaster Food Soc..

[26] Nakakuki K., Nakakuki K., Kitahara M., Ando Y. (2015). Purpose and significance of dental support activities in natural disaster. Dental Health Care Measures in Case of Disaster―For Cooperation and Standardization.

[27] Moriwaki H., Kashima H., Moriwaki H. (2020). Aging. Public Health Nutrition for Wellness.

[28] Centre for Risk Studies (2014). The Cambridge World City Risk Atlas.

[29] Munakata E. (2017). Make use of women’s power for disaster prevention: Research on “earthquake disaster and women” with the Great East Japan Earthquake. Jpn. J. Fam. Relat..

[30] Ohara M. (2012). Issues on disaster emergency response from the perspective of gender equality. Mon. J. Inst. Ind. Sci. Univ. Tokyo.

[31] Schmitt M.L., Clatworthy D., Ratnayake R., Klaesener-Metzner N., Roesch E., Wheeler E., Sommer M. (2017). Understanding the menstrual hygiene management challenges facing displaced girls and women: Findings from qualitative assessments in Myanmar and Lebanon. Confl. Health.

[32] Cabinet Office (Disaster Management) Shelter Management Guidelines. http://www.bousai.go.jp/taisaku/hinanjo/pdf/1604hinanjo_guideline.pdf.

[33] Anzai Y., Katsura S., Bando S., Kawarabata N., Chiba Y., Nihei E., Ono S. (2018). Experiences of elderly refuge residents after the Great East Japan Earthquake and tsunami:—Focusing on the post-earthquake acute disaster phase. Jpn. J. Public Health Nurs..

[34] Takahashi S., Kuwahara Y., Matsui Y. (2014). Acute stress reaction of the disaster-affected local governments’ employees by the Great East Japan Earthquake. Stress Sci. Res..

[35] Beppu S. (2019). Disaster food, a new approach in terms of food in a period of most natural disaster events. J. Cook. Sci. Jpn..

[36] Kotorii A., Koda H., Sudo N., Tsuchida N., Doi K., Beppu S., Mashiro G., Mori S., Mori M. (2018). A web-based questionnaire survey of stockpiled foods which support the implementation of business continuity plan in companies. J. Jpn. Disaster Food Soc..

[37] Mizutani Y. (2012). Supply methods of corrugated box portable bed to refuges in disaster and an action of disaster prevention agreement in Japan. Jpn. J. Phlebol..

[38] Labaš D. (2017). The impact of organizational crisis preparedness on firm business performance. Mark. Trz..

[39] Kesete Y., Peng J., Gao Y., Shan X., Davidson R.A., Nozick L.K., Kruse J. (2014). Modeling insurer-homeowner interactions in managing natural disaster risk. Risk Anal..

[40] Peng J., Shan X.G., Gao Y., Kesete Y., Davidson R.A., Nozick L.K., Kruse J. (2014). Modeling the integrated roles of insurance and retrofit in managing natural disaster risk: A multi-stakeholder perspective. Nat. Hazards.

[41] Shan X., Peng J., Kesete Y., Gao Y., Kruse J., Davidson R.A., Nozick L.K. (2017). Market Insurance and Self-Insurance through Retrofit: Analysis of Hurricane Risk in North Carolina. ASCE-ASME J. Risk Uncertain. Eng. Syst. Part A Civ. Eng..

[42] McManus D.J., Carr H.H. (2000). Risk and the need for business continuity planning. Business Continuity Planning: Protecting Your Organization’s Life.

